# Oxidative stress vs hormonal profile in plasma and saliva: application in sport performance

**DOI:** 10.1186/1550-2783-8-S1-P34

**Published:** 2011-11-07

**Authors:** Fabrizio Angelini, Danieala Buonocore, Sara Rucci, Gianulca Stesina, Luca Stefanini, Allesandro Bonuccelli, Fabrizio Tencone, Fulvio Marzatico

**Affiliations:** 1 Society of Sport Nutrition and Wellness; 2 Medical Staff, Juventus FC, Turin, Italy; 3 Laboratory of Pharmacobiochemistry, Nutrition and Nutriceuticals of Health, University of Pavia, Pavia, Italy

## 

Oxidative stress, a condition defined as unbalancing between production of free radicals and antioxidant defenses, is an important index of health status to monitor wellness and sport performance. Today, many aspects of hormone role in regulating oxidant – antioxidant balance still remain obscure. Physical and psychological stressor, which activate pituitary-adrenal axis, cause oxidative damage (Mancini et al., 2010).Oxidative stress and inflammation are traditionally associated with fatigue and impaired recovery from exercise and antioxidant could play a positive role to reduced inflammation markers and cortisol response (Tidus et al., 1995). Furthermore a relationship between sex hormones and plasmatic Total Antioxidant Capacity (TAC) was observed. TAC is significantly correlated with total testosterone in male subjects (Mancini et al., 2010). Aim of this work is to obtain first data which correlate plasmatic oxidative stress (TAC and lipid peroxidation) with levels of testosterone and cortisol (T/C),recommended as good markers of training stress (Banfi et al., 1993), during season of a top team of the Italian Soccer League. Furthermore during the same season we assessed the same levels of testosterone and cortisol in saliva and correlated them with obtained data in plasma. To evaluate oxidative stress in plasma we used two validated techniques OXY-Adsorbent and d-ROMs test. The first one measures plasma TAC against a massive oxidative insult induced *in vitro* by a hypochlorous acid solution while d**-ROMs test** measures lipid peroxides amount produced by ferrous iron solution action.Our data indicate that there is no correlation between TAC and d-ROMs showing them as the best marker for oxidative stress. There is a correlation between T/C databoth in plasma and saliva with d-ROMs. T/C *Ratio* decrease from July to January and remainsroughlystable, with aminimumincreasein April both in plasma and saliva. It’s an important result that validate the possibility to assess hormone levels in both physiological fluids and confirm that saliva can be used as an alternative non invasive method to evaluate hormonal levels.

